# Damaging coding variants within kainate receptor channel genes are enriched in individuals with schizophrenia, autism and intellectual disabilities

**DOI:** 10.1038/s41598-019-55635-4

**Published:** 2019-12-16

**Authors:** Maria Koromina, Miles Flitton, Alix Blockley, Ian R. Mellor, Helen M. Knight

**Affiliations:** School of Life Sciences, University of Nottingham, Nottingham, NG7 2UH UK

**Keywords:** Medical genetics, Ion channels in the nervous system

## Abstract

Schizophrenia (Scz), autism spectrum disorder (ASD) and intellectual disability are common complex neurodevelopmental disorders. Kainate receptors (KARs) are ionotropic glutamate ion channels involved in synaptic plasticity which are modulated by auxiliary NETO proteins. Using UK10K exome sequencing data, we interrogated the coding regions of KAR and NETO genes in individuals with Scz, ASD or intellectual disability and population controls; performed follow-up genetic replication studies; and, conducted *in silico* and *in vitro* functional studies. We found an excess of Loss-of-Function and missense variants in individuals with Scz compared with control individuals (*p* = 1.8 × 10^−10^), and identified a significant burden of functional variants for Scz (*p* < 1.6 × 10^−11^) and ASD (*p* = 6.9 × 10^−18^). Single allele associations for 6 damaging missense variants were significantly replicated (*p* < 5.0 × 10^−15^) and confirmed *GRIK3* S310A as a protective genetic factor. Functional studies demonstrated that three missense variants located within GluK2 and GluK4, GluK2 (K525E) and GluK4 (Y555N, L825W), affect agonist sensitivity and current decay rates. These findings establish that genetic variation in KAR receptor ion channels confers risk for schizophrenia, autism and intellectual disability and provide new genetic and pharmacogenetic biomarkers for neurodevelopmental disease.

## Introduction

Schizophrenia and autism are common, highly heritable debilitating neurodevelopmental disorders which are often comorbid with intellectual disability. Advances in genomic technology suggest a role for both common and rare variants contributing to genetic risk^1–4^. For instance, whole genome and exome sequencing studies have indicated that ultra-rare Loss-of-Function (LoF) point mutations and indel and CNV variants that truncate proteins, are enriched in individuals with neuropsychiatric disease and are both inherited and *de novo*^5–9^. Such studies commonly find that the disrupted genes are involved in synaptic function, which has led to the increasingly used term ‘synaptopathy’ for brain disorders that are thought to arise from synaptic dysfunction^10–12^.

Kainate receptors (KARs) are ionotropic glutamatergic receptors which form functional ion channels by tetrameric combinations of five different subunits. GluK1-3 subunits form functional homomeric or heteromeric receptors while GluK4 and GluK5 participate as functional receptors when combined with GluK1-3 subunits. Each subunit consists of an extracellular amino terminal domain, an extracellular ligand binding domain, three transmembrane domains and a loop M2, and a C-terminal domain. Mutating amino acids in these conserved regions gives rise to changes in channel properties^13^ and can alter the affinity for external cations such as sodium and lithium^14,15^. KAR channel properties are also regulated by the presence of the KAR auxiliary subunits Neuropilin And Tolloid Like 1, NETO1, and Neuropilin And Tolloid Like 2, NETO2^16–18^.

KARs contribute to generating post-synaptic excitatory responses and to shorter term synaptic plasticity mechanisms by influencing presynaptic transmitter release^19^. In recent years, their action in non-canonical G protein coupled signalling, new forms of Long Term Potentiation, and synaptic targeting mechanisms have also been recognised^20–23^. NETO1 and NETO2 are single pass transmembrane CUB domain-containing proteins which influence the trafficking of KAR subunits and kinetics of KAR function^24^. During development KARs and Netos are highly expressed in the brain and are important for synaptogenesis, neurite outgrowth and glutamatergic pathway connectivity^25–27^. Rodent knock out and transgenic models have provided some evidence that loss of these receptors influences brain function and behaviour in a manner analogous to human disease^28–33^. In humans, common, non-coding, intronic variants and private *de novo* mutations located within specific GluK subunit genes (e.g. *GRIK1*, *GRIK2*, *GRIK3*, *GRIK4, GRIK5*) have been associated with a broad spectrum of neurological diseases^34–39^ and GluK genotype-dependent changes in cognition, brain activation and response to antidepressant and antipsychotic treatments have been reported^40–43^. However, as yet no comprehensive screen of coding variants across all GluK genes or KAR auxiliary proteins have been performed in cohorts with intellectual disability and neurodevelopmental conditions.

We hypothesize that damaging coding alleles within KAR subunit and NETO genes contribute to risk for developing neurodevelopmental disorders. Here we characterised LoF variants, performed single allele association and burden enrichment analysis of damaging coding variants within KAR subunit and NETO genes, in individuals with schizophrenia, psychosis, autism and intellectual disabilities available from the UK10K project and ExAC study^44^. We subsequently examined the functional effect of predicted damaging missense variants within GluK2 and GluK2/GluK4 receptors using *in silico* modelling tools and *in vitro* electrophysiological assays.

## Results

### LoF and rare missense variants are increased within individuals with neurodevelopmental disorders

**Figure 1:**
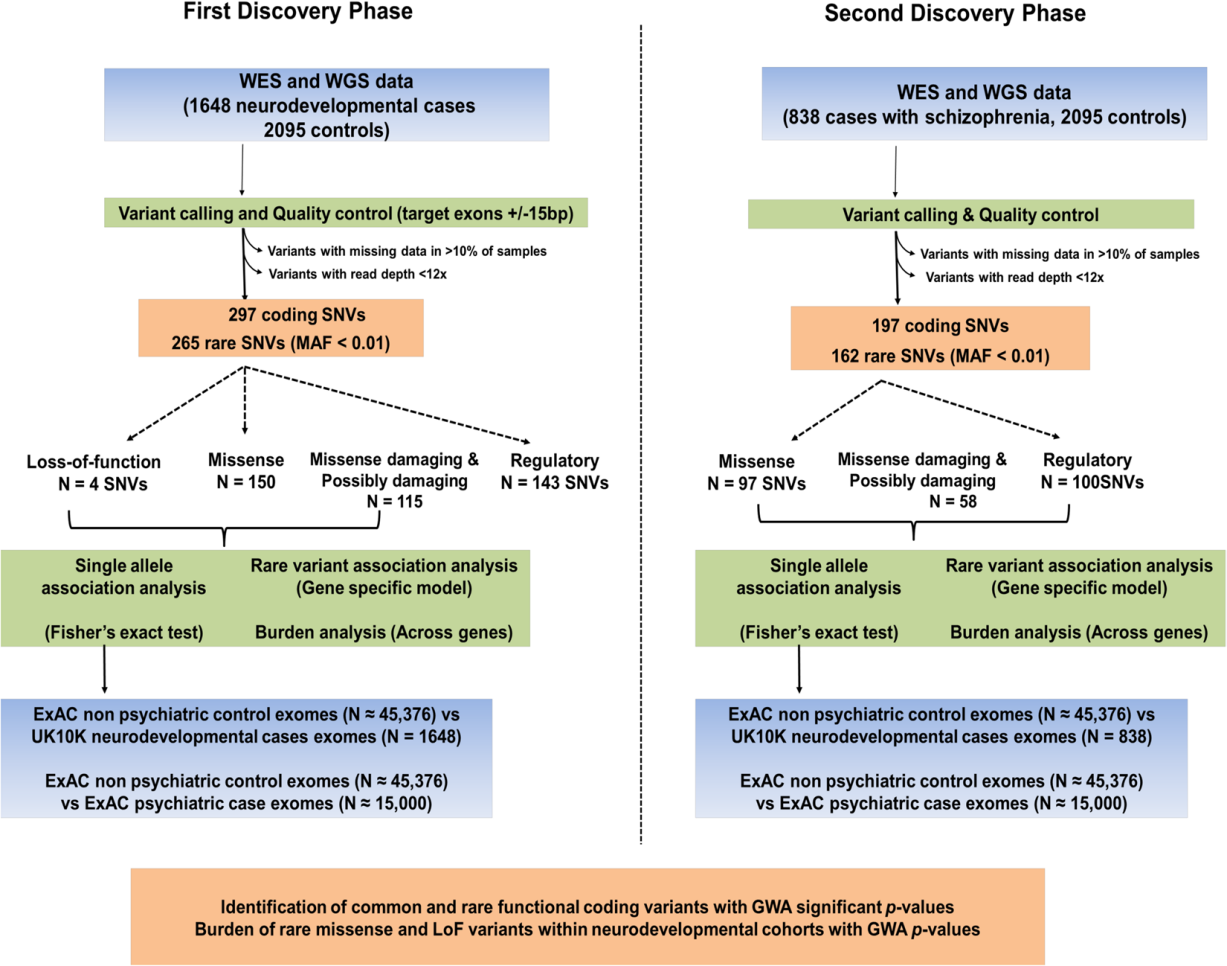
Genetic analysis workflow. Data sets investigated are shown in blue, methods and analysis in green and findings are colored orange. Discovery phase one analyzed WES and WGS from cohorts with neurodevelopmental disorders (including severe neuropsychiatric conditions and ASD, ID and dual Scz-ID) and general population control cohorts. The second discovery phase investigated two additional schizophrenia cohorts. Details of the cohorts are presented in Supplementary Table S1. Single allele associations and burden analysis was performed. Associated alleles were followed up by performing case-control studies using the ExAC cohort non psychiatric control and psychiatric case populations. Abbreviations: ASD, autism spectrum disorders; ExAC, Exome Aggregation Consortium; FINSCZ, Finnish schizophrenia samples; ID, Intellectual Disability; MAF, Minor Allele Frequency; Scz, schizophrenia; SNV, Single-Nucleotide Variant; WES, Whole-Exome Sequencing; WGS, Whole-Genome Sequencing.

The pipeline followed for the genetic analysis is presented in Fig. 1. In a first discovery phase, we identified 154 non-synonymous variants which included 4 LoF and 150 missense variants and 143 regulatory variants within *GRIK1-5* and *NETO1-2* genes (Table 1). 265 variants had a MAF < 1% and were classified as rare or ultra-rare. We postulated that genes, which in the general population, are characterised as having few LoF and missense variants would carry high numbers of LoF and damaging variants in individuals with neurodevelopmental disorders as these variants putatively contribute to disease risk. In the ExAC project database, *GRIK2, GRIK3, GRIK5* and *NETO1* are all classified as LoF intolerant genes (LoF pLI > 0.90) indicating that these genes are extremely intolerant of Loss-of-Function variation and hence few LoF mutations are present in the general population. As detailed in Table 1, we identified 4 LoF variants within affected individuals who had either ID or ASD or comorbid with Scz *(GRIK1* L411X, Scz comorbid with ID; *GRIK4* S98X, ID; *GRIK5* Q848X, ASD; and *GRIK5* 19:42546908 splice acceptor, variant, ASD), and two of which were within the LoF intolerant gene *GRIK5*. No LoF variants were identified in the control cohort.

**Table 1:** LoF and damaging missense variants identified in the discovery and replication phases as significantly associated with disease risk*.* Variant location, type of variant, MAF and allele counts for affected cases, controls and population controls (ExAC cohort), diseases diagnosis, *p*-values for association and Odds Ratios are presented. Abbreviations: ASD, Autism spectrum disorder; ExAC, Exome Aggregation Consortium; ct, (allele) count; Diag, diagnosis; Gen ct, genotype count; ID, intellectual disability; Inf, infinity; MAF, Minor Allele Frequency; Mis, missense variant; Non, nonsense mutation; OR, Odds Ratio; PSC, premature start codon gain; Scz, schizophrenia; Scz-ID, dual diagnosis of schizophrenia and ID; SpA, splice acceptor variant; SpI, splice intronic variant.

Gene	cDNA	Type	MAF cases (Gen ct)	Diag	MAF con (Gen ct)	*P* value	OR (CI)	MAF ExAC
FIRST DISCOVERY PHASE								
*GRIK1*	c.1232 T > A (p.Leu411*)	Non	3.03 × 10^−4^ (1 T/A)	Scz-ID			Inf	5.78 × 10^−5^
*GRIK3*	c.928 T > G (p.Ser310Ala)	Mis	0.17 (451 T/G, 51 G/G)	All	0.25 (846 T/G, 113 G/G)	1.01 × 10^−18^	0.59 (0.52–0.66)	0.27
*GRIK3*	c.1756T > G (p.Phe586Val)	Mis	0.003 (9 T/G)	ASD		2.84 × 10^−7^	Inf	Novel
*GRIK4*	c.293 C > A (p.Ser98*)	Non	3.03 × 10^−4^ (1 C/A)	ID			Inf	Novel
*GRIK5*	c.2542 C > T (p.Gln848*)	Non	3.03 × 10^−4^ (1 C/T)	ASD			Inf	Novel
*GRIK5*	c.2684 C > G (p.Ala895Gly)	Mis	0.005 (9 C/G, 4 G/G)	Scz		4.06 × 10^−5^	44.83 (2.70–765)	Novel
*GRIK5*	c.1270-1 G > T	SpA	3.03 × 10^−4^ (1 G/T)	ASD			Inf	Novel
*NETO1*	c.-143G > T	PSC	3.03 × 10^−4^ (1 G/T)	ASD			Inf	Novel
SECOND DISCOVERY PHASE								
*GRIK2*	c.1525–10 C > T	SpI	0.010 (16 C/T)	Scz		4.43 × 10^−8^	42.26 (2.53–704)	Novel
*GRIK3*	c.2593 A > G (p.Arg865Gly)	Mis	0.008 (14 A/G)	Scz		6.81 × 10^−6^	73 (4–1226)	0.005
			**MAF (ct) Cases**	**MAF (ct) Con ExAC**		***P*** **value**	**OR (CI)**	
ExAC REPLICATION FOLLOW UP (1)								
*GRIK1*	c.2705 T > C (p.Leu902Ser)	Mis	0.031 (101/3,288)	0.08 (68,18/90,756)		4.83 × 10^−15^	0.399 (0.320–0.476)	
*GRIK3*	c.928 T > G (p.Ser310Ala)	Mis	0.168 (553/3,288)	0.29 (25,607/90,756)		6.49 × 10^−50^	0.513 (0.469–0.564)	
*GRIK5*	c.2684 C > G (p.Ala895Gly)	Mis	0.005 (17/3,288)	0.00 (0/90,756)		8.55 × 10^−24^	Inf	
			**MAF (ct) Psy ExAC**	**MAF (ct) Con ExAC**		***P*** **value**	**OR (CI)**	
ExAC REPLICATION FOLLOW-UP (2)								
*GRIK1*	c.2705 T > C (p.Leu902Ser)	Mis	0.040 (1,155/30,000)	0.075 (6,818/90,756)		1.145 × 10^−108^	0.49 (0.46–0.53)	
*GRIK3*	c.928 T > G (p.Ser310Ala)	Mis	0.245 (7,364/30,000)	0.29 (25,607/90,756)		4.076 × 10^−35^	0.81 (0.80–0.84)	
*GRIK4*	c.1582 G > A (p.Val528Ile)	Mis	0.0002 (5/30,000)	0.011 (1,028/90,756)		5.467 × 10^−74^	0.01 (0.004–0.03)	
*NETO1*	c.1460 C > G (p.Ala487Gly)	Mis	6.60 × 10^−5^ (2/30,000)	0.008 (704/90,756)		7.967 × 10^−52^	0.009 (0.001–0.03)	
*NETO2*	c.1366 T > A (p.Ser456Thr)	Mis	6.60 × 10^−5^ (2/30,000)	0.009 (808/90,756)		2.056 × 10^−59^	0.007 (0.001–0.02)	

**Figure 2:**
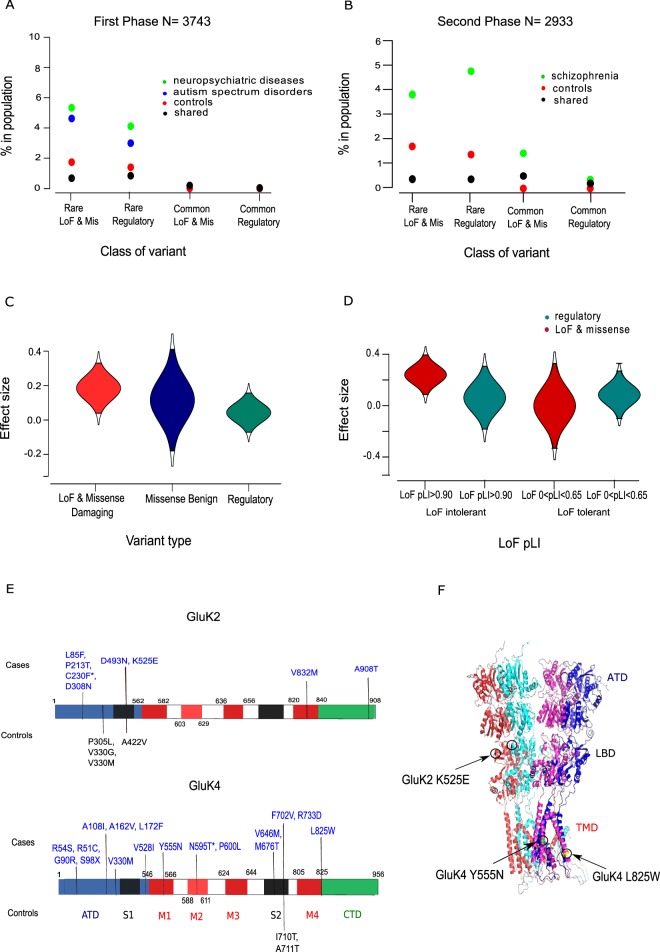
Frequency and effect size of functional variants and the location of non-synonymous variants within *GRIK2* and *GRIK4*. (**A**) Individuals with severe neuropsychiatric disease (i.e. conditions with psychosis) and ASD have a higher percentage of rare functional variants compared to control individuals in the first discovery study. (**B**) Individuals with schizophrenia have a have a higher percentage of rare functional variants compared to control individuals in the second discovery study. (**C**) LoF and missense variants have a larger risk effect size than regulatory variants in genes which are classified as LoF and missense intolerant. (**D**) Risk effect sizes are smaller for regulatory variants and benign missense variants compared to LoF and damaging missense variants. (**E**) The location of LoF and damaging missense variants identified within *GRIK2* and *GRIK4* proteins in affected and control individuals. Protein domains are colour coded with the ATD as blue; LBDs (S1 and S2) as black; transmembrane domains M1-M3-M4 red; M2 loop as red and the C-terminal domain (CTD), green. Damaging missense variants carried in cases or controls or are colour coded as blue and black respectively. (**F**) The location of three damaging missense variants, GluK2 K525E, GluK4 Y555N, and GluK4 L825W. The term ‘shared’ denotes variants found within both case and control groups. Abbreviations: ATD, amino terminal domain; LoF pLI, propability for LoF intolerance score.

ExAC also categorizes *GRIK2*, *GRIK3*, *GRIK4*, *GRIK5* and *NETO1* as missense intolerant genes (missense z > 2.80) again suggesting that fewer missense mutations are present in the general population within these genes than expected. Of the 150 missense variants identified in the current study, 75 were considered as protein damaging, 40 as possibly damaging variants and 35 as benign. The number of rare and ultra-rare nonsynonymous variants within affected individuals was found to be higher than the number of rare nonsynonymous variants found within control individuals or shared despite the fact that the control population has twice the number of individuals (affected case frequency 0.58; control frequency 0.26; variants found in both cases and controls frequency 0.16) (Supplementary Tables S4–S7). As indicated in Fig. 2, the majority of LoF and predicted damaging missense variants were identified in individuals with Scz and ASD whilst most of the predicted benign missense variants were found either in controls or were shared between cases and controls subjects (Supplementary Table S8). In contrast to rare LoF and missense mutations, an equal frequency of common non-synonymous, synonymous and regulatory variants was found in both case and control individuals. The most common variants identified (MAF > 0.1) were synonymous variants (87.5%) and present in both case and control individuals.

We also found that the effect size of rare LoF and missense variants within LoF intolerant genes (LoF pLI > 0.90), was larger than the effect size (0.3 compared to −0.2) of rare LoF and missense variants within genes with a low LoF tolerance metric (Fig. 2d). In addition, as shown in Fig. 2 and Supplementary Fig. S1, rare LoF and missense variants were indicated to have a larger effect size compared to variants classified as regulatory and the effect size of rare regulatory variants did not differ between LoF intolerant and LoF tolerant genes.

### Single coding alleles associated with risk or protection against developing disease

Two single coding variants were found to be significantly associated with disease (Table 1 and Supplementary Figs. S2, S3). *GRIK3* S310A, located in the amino terminal domain (ATD), was found to be protective against a broader neurodevelopmental phenotype, e.g. conditions with psychosis and ASD/ID phenotypes (*p* = 1.01 × 10^−18^; OR = 0.59, CI 0.52–0.66,). *GRIK3* F586V, was associated with risk of developing ASD (*p* = 2.84 × 10^−7^; OR = Inf). In addition, A895G located within the cytoplasmic protein domain of *GRIK5*, was found protective at a nominal level of significance against developing schizophrenia (*p* = 4.06 × 10^−5^; OR = 44.83, CI 2.70–765).

### Enrichment of coding variants within *GRIK* and *NETO* genes

We analysed the burden and accumulation rates of functional variants and found a significantly increased burden of LoF, missense and regulatory variants which included all allelic frequencies in the case population (*p* = 3.38 × 10^−20^). Similarly, and as detailed in Table 2, we also found an increased burden of ultra-rare and rare LoF, missense and regulatory variants (*p* = 2.07 × 10^−15^). When comparing a broader neurodevelopmental phenotype, e.g. ASD, psychosis and ID, or a narrower psychosis phenotype with control individuals, we observed a significantly increased burden of LoF and missense variants within all *GRIK* and *NETO* genes (broad neurodevelopmental, all allele frequencies, *p* = 2.97 × 10^−10^; broad neurodevelopmental, ultra-rare and rare *p* = 6.02 × 10^−7^; psychosis, all frequencies; *p* = 6.17 × 10^−7^; psychosis, ultra-rare and rare *p* = 1.83 × 10^−7^). We also found a significantly increased burden of LoF, missense and regulatory variants at all allele frequencies (*p* = 6.86 × 10^−18^) and for ultra-rare and rare variants alone (*p* = 1.30 × 10^−9^) for the combined intellectual disability/ASD cohorts. However, although we found a significant burden of common and rare variants (*p* = 3.15 × 10^−11^) for ASD/ID, ultra-rare and rare variants alone did not reach genome-wide level of significance (*p* = 0.026).

**Table 2:** Enrichment of variants within *GRIK* and *NETO* genes associated with neurodevelopmental disorders. LoF, missense and regulatory variants were analyzed by study phase and grouped by allele frequency and diagnosis. SKAT-O *p* values indicate a significant enrichment of risk and protective variants in affected individuals. Abbreviations ASD, Autism spectrum disorder; ID, intellectual disability.

Variant Type	Frequency	Disease status	*P* value (SKAT- O)
FIRST DISCOVERY STUDY (1648)			
All functional	All (0.0–0.50)	All neurodevelopmental	3.38 × 10^−20^
		Psychosis	1.63 × 10^−11^
		ASD & ID	6.86 × 10^−18^
	Utra rare & rare (<0.01)	All neurodevelopmental	2.07 x 10^−15^
		Psychosis	3.69 × 10^−13^
		ASD & ID	1.30 × 10^−9^
LoF and missense	All (0.0-0.50)	All neurodevelopmental	2.97 × 10^−8^
		Psychosis	6.17 × 10^−7^
		ASD & ID	3.15 × 10^−11^
	Ultra rare & rare (<0.01)	All neurodevelopmental	6.02 x 10^−7^
		Psychosis	1.83 × 10^−10^
		ASD & ID	0.026
Regulatory	All (0.0–0.50)	All neurodevelopmental	3.37 x 10^−6^
		Psychosis	1.83 × 10^−7^
		ASD & ID	6.20 × 10^−4^
	Ultra rare & rare (<0.01)	All neurodevelopmental	1.17 × 10^−6^
		Psychosis	1.83 × 10^−7^
		ASD & ID	6.06 x 10^−6^
SECOND DISCOVERY STUDY (838)			
All functional	All (0.0–0.50)	Schizophrenia	1.26 × 10^−25^
	Utra rare & Rare (<0.01)	Schizophrenia	3.55 × 10^−7^
LoF and missense	All (0.0–0.50)	Schizophrenia	4.39 × 10^−15^
	Utra rare & Rare (<0.01)	Schizophrenia	0.138
Regulatory	All (0.0–0.50)	Schizophrenia	7.37 × 10^−22^
	Utra rare & Rare (<0.01)	Schizophrenia	2.10 × 10^−14^

Burden analysis was also performed for each individual gene (Supplementary Tables S9–S11). *GRIK3*, *GRIK5* and *NETO1*, three genes classified as LoF and missense intolerant, were indicated as having an increased burden of functional variants (common and rare variants combined for *GRIK3 p* = 1.26 × 10^−5^; *NETO1 p* = 4.66 × 10^−16^; and rare variants only for *GRIK5 p* = 9.99 × 10^−6^). We also assessed variant load *per* gene level grouped by either severe neuropsychiatric phenotypes, i.e. conditions with psychosis, or ASD/ID phenotypes. For the psychosis grouping, we observed a genome-wide significant burden of rare LoF and missense variants within *GRIK5* (*p* = 7.83 × 10^−10^) and an increased burden of common and rare functional variants within *NETO1* (*p* = 6.76 × 10^−6^). For ASD and ID samples, we identified a genome-wide or nominal significant burden of common and rare LoF, missense and regulatory variants within *GRIK3* (*p* = 3.31 × 10^−13^), *GRIK1* (*p* = 1.20 × 10^−5^) and *NETO1* (*p* = 2.79 × 10^−12^).

### *GRIK* and *NETO* genetic associations in two additional schizophrenia cohorts

In a second discovery phase, we investigated the exomes of two additional schizophrenia datasets, the UKSCZ (N = 553) and FSZNK (N = 285) cohorts. Unlike in the first discovery phase where LoF variants were identified in individuals with ID or ASD, we did not identify any LoF variants in this second phase. This may relate to the fact that all affected individuals had a diagnosis of Scz and not ID or ASD. However, we detected 197 coding variants of which 97 were missense variants. 58 missense variants were predicted damaging and 34 (59%) of these damaging missense variants were identified within affected individuals only (Supplementary Tables S12–S16). As before, we investigated *GRIK* and *NETO* single coding alleles for association with risk or protection against Scz (Figs. S1–S3). A splice variant within *GRIK2* 6:102337505, c.1525–10 C > T (*p* = 4.43 × 10^−8^; OR = 42.26, CI 2.53–704) and a missense variant within *GRIK3*, R865G, (*p* = 6.8 × 10^−6^; OR = 73, CI 4–1226) showed a significant nominal association with risk for schizophrenia (Table 1).

Consistent with our previous findings for a psychosis phenotype, we observed that *GRIK* and *NETO* genes had high accumulation rates of functional coding variants (Table 2, Supplementary Table S17). For instance, we found a significantly increased burden of missense and regulatory variants at all allele frequencies (*p* = 1.26 × 10^−25^), and a significantly increased burden of just missense variants (*p* = 4.39 × 10^−15^). In addition, a higher burden of common and rare missense variants were found within *GRIK3* (*p* = 5.24 × 10^−10^) and both *NETO1* and *NETO2* genes had higher accumulation rates of common and rare regulatory variants (*p* = 1.60 × 10^−28^ and *p* = 8.03 × 10^−10^). We also observed a burden of rare regulatory variants within *GRIK5* (*p* = 3.37 × 10^−5^).

### Evidence for the robustness of allele associations

To assess the robustness of significantly associated single alleles identified in discovery phases, we compared allelic frequencies of rare and common coding or splicing variants in the UK10K discovery phase affected cases with non-affected exomes from ExAC cohorts (N = 45,376). We identified 8 genome-wide significant associations (3 missense, 4 synonymous and 1 splice site) listed in Table 1 and Supplementary Table S18 validating our previous findings. Of the 3 missense variants, two had significant associations for protection against neuropsychiatric disease, *GRIK3* S310A (*p* = 6.49 × 10^−50^; OR = 0.51, CI 0.47–0.56) and *GRIK1* L902S (*p* = 4.83 × 10^−15^; OR = 0.40, CI 0.32–0.48), whilst one showed a significant association for risk for neuropsychiatric disease, *GRIK5* A895G (*p* = 8.55 × 10^−24^; OR = Inf). We also identified 6 variants associated at the nominal level of significance (3 missense, 1 synonymous and 2 splice site variants), Supplementary Table S18.

Finally, using data from ExAC for well individuals (N = 45,376) and from the psychiatric disease arm of the ExAC study (N = 15,328) which includes individuals with additional neurological and psychiatric conditions, e.g. Tourette’s syndrome, and hence relates to a yet broader neurodevelopmental phenotype, we compared allele frequencies for all damaging coding variants which we had previously identified in the earlier phases of the study. We observed nine significant associations (*p* < 2 × 10^−34^); 5 missense variants, 3 synonymous and one splice variant, details of which are provided in Table 1 and Supplementary Table S19. The levels of significance (1 × 10^−6^ to 1 × 10^−108^) reflects the power to detect associations with very large sample numbers, e.g. N = 45,000, and is consistent with values reported for individual variants studied in large cohorts^44–46^.

Consistent with our previous findings, we again observed a significant difference in allele frequencies for the *GRIK3* S310A variant (non-affected individuals MAF 0.29, disease MAF 0.25; *p* = 4.07 × 10^−35^; OR 0.81, CI = 0.80–0.84) and nominal significance for *GRIK3* R865G variant (non-affected individuals MAF 0.004, disease MAF 0.006; *p* = 2.07 × 10^−5^; OR 1.49, CI = 1.22–1.73).

### *In silico* and *in vitro* assays of rare variants within GluK2 and GluK4 TMD and LBD domains support a functional effect

Three predicted damaging missense mutations identified in individuals with schizophrenia and located within ‘key’ ligand binding (GluK2 K525E) and transmembrane (GluK4 L825W; GluK4 Y555N) domains of GluK2 and GluK4 subunits, were examined using *in silico* modelling tools. We found that GluK4 Y555N disrupted a hydrogen bond and resulted in a significant destabilizing thermodynamic effect (∆∆G = 1.65). GluK4 L825W did not affect the formation of hydrogen bonds but did however, have a slightly destabilizing effect on the total energy (∆∆G = 0.755). The GluK2 LBD K525E mutation led to creation of a hydrogen bond but no predicted observable thermodynamic effect was predicted (∆∆G = 0.06), shown in Supplementary Figure S4. However, a decrease in predicted positive electrostatic potential was observed over the ligand binding domain area of the GluK2 K525E variant (Supplementary Figure S5) and which could influence cell surface expression^47^. Taken together, *in silico* protein modeling analysis suggests that these three predicted damaging mutations could affect protein conformation, structural relationships or electrostatic potential.

**Figure 3:**
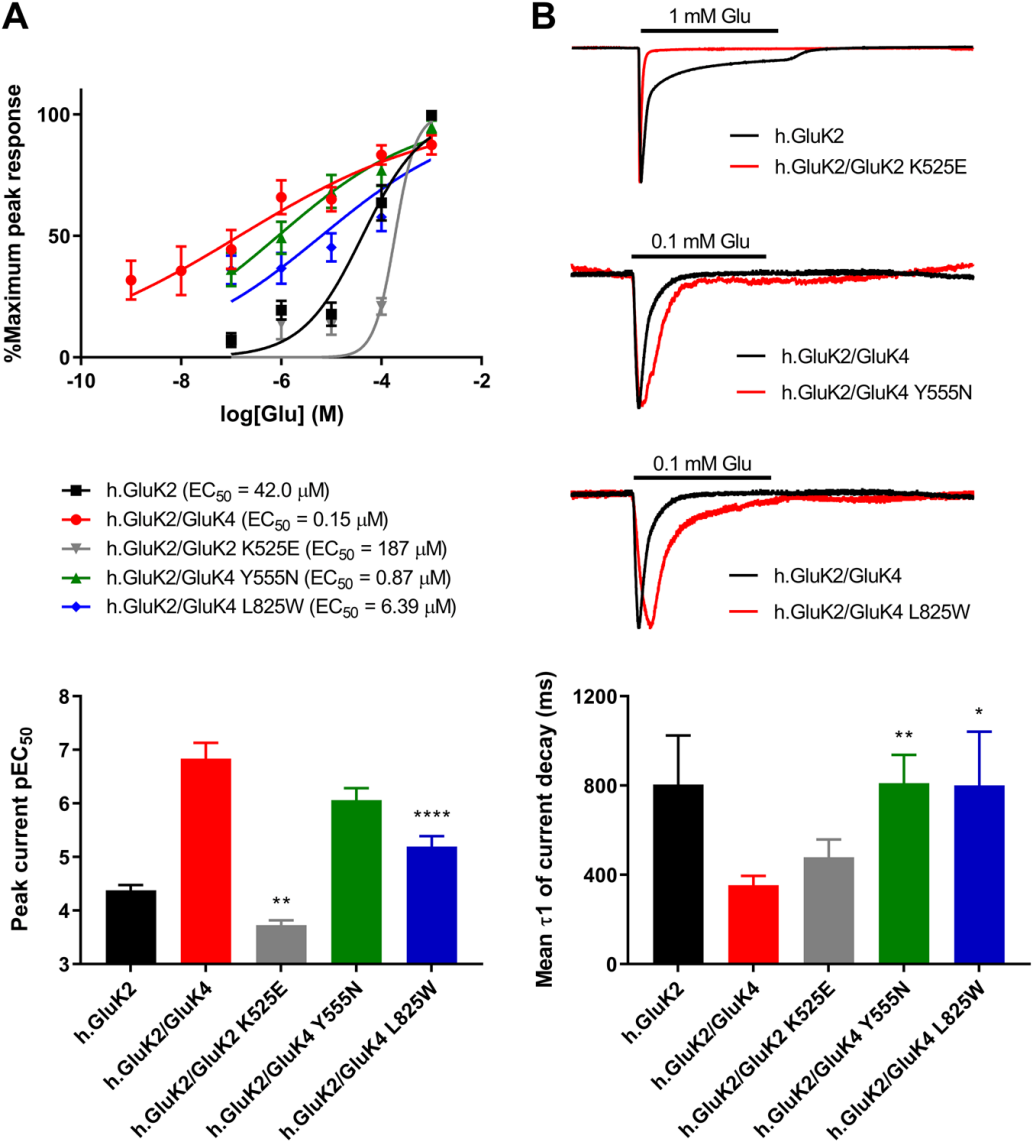
Altered channel properties of GluK2 and GluK4 mutants. (**A**) Top: Glutamate concentration-response curves for wild type GluK2 and GluK2/GluK4 receptors and the mutated GluK2/GluK2 K525E, GluK2/GluK4 Y555N and GluK2/GluK4 L825W subunit combinations. Points are mean % of maximum peak response ± SEM and curves are fits of the Hill equation. Bottom: Comparisons of pEC_50_ for wild type and mutated GluK2 K525E, GluK4 Y555N, GluK4 L825W KARs. (**B**) Top: Two microelectrode voltage clamp (TEVC) traces for wild type and mutant GluK2 K525E, GluK4 Y555N, GluK4 L825W KARs in response to a 10-s application (bar) of 1 mM glutamate (for wild type and mutated GluK2 receptors) or 0.1 mM glutamate (for wild type and mutated GluK2/GluK4 receptors) at −80 mV. The responses have been scaled such that the peak responses are equal to aid comparison of the current decay kinetics, hence there are no vertical scale bars. Bottom: Comparisons of current decay τ1 values for wild type and mutated GluK2 K525E, GluK4 Y555N, GluK4 L825W KARs. Statistically significant differences are indicated by *(*p* < 0.05), **(*p* < 0.01) or ****(*p* < 0.0001). Abbreviations: Glu, glutamate; hGluK2, human GluK2; KA, kainic acid, pEC_50_: negative logarithm of the EC_50_ value.

To further study functional changes, we expressed wild-type and mutated GluK2 homomers and GluK2/GluK4 heteromers in *Xenopus* oocytes and measured their current responses to application of glutamate using a voltage-clamp. Currents rose to a peak then decayed to a steady state (Fig. 3b). Because of the temporal limitations of this system, currents represent a mixture of activation, desensitization and deactivation processes but presumably with activation dominating the rising phase and desensitization and deactivation dominating the decaying phase. We measured the agonist sensitivity of the peak current (EC_50_) and the current decay rates (τ_decay_) and although these current kinetics are not physiological, we compared changes in responses between wild-type and mutant receptors (Fig. 3; Supplementary Table 20).

GluK4 L825W reduced glutamate potency of peak current generation for GluK2/GluK4 receptors by 42.6 -fold (*p* = 0.0001, n = 10–18). Similarly, the GluK4 Y555N mutant decreased glutamate potency for GluK2/GluK4 receptor peak currents by 15 -fold (*p* = 0.0001, n = 9–13). The GluK2 K525E LBD variant was not functional by itself but when co-expressed with GluK2, glutamate potency of GluK2 homomers was decreased by 4.5-fold (*p* < 0.05, n = 15–17) (Fig. 3a, b). These results imply decreased KAR channel activity.

The rate of current decay of GluK2/GluK4 L825W and GluK2/GluK4 Y555N heteromers was found to be 2.3-fold slower (*p* = 0.047 and *p* = 0.002 respectively) than for GluK2/GluK4 wild type receptors upon application of 0.1 mM glutamate (Fig. 3, Supplementary Table S20). This would suggest that both GluK4 mutations may result in mildly increased function through the channels potentially remaining open for a prolonged time. GluK2 K525E did not significantly affect the rate of current decay. Taken together, our observations support that there is likely an overall decreased function in all mutants.

## Discussion

Our findings from an integrated analysis of ~4,580 genomes investigated in two discovery phases, supports the hypothesis that LoF and damaging variants within KAR subunit and NETO genes are enriched in individuals with schizophrenia, autism and ID. Our observations of a specific candidate gene set are congruent with recent large scale whole genome and exome studies of individuals with schizophrenia and schizophrenia with ID which report an increased burden of ultra-rare coding and common variants in genes characterised as missense and LoF variant depleted genes^9,48^. Likewise, our findings provide further support that, in addition to rare *de novo* variation as a strong causative factor for autism^5,49^, inherited LoF and damaging mutations can confer risk for autism and ID. We also confirm evidence that Scz, ASD and ID phenotypes share genetic predisposing factors and neuropathology^50–52^, and that variants with a spectrum of allele frequencies and effect size within *GRIK* and *NETO* genes contribute to these phenotypes.

We identified LoF and damaging missense variants across key protein domains of KAR subunits involved in specific functions. The majority of the significantly associated replicated alleles were found to be protective, e.g. *GRIK3* S310A and are novel targets for future genetic studies. Our electrophysiological findings supported the idea that both LBD and TMD mutants altered channel gating behavior. However, variants may also impact upon KAR function by a number of alternative means. For instance, disruption of KAR and NETO interaction may affect the synaptic localisation of KARs^53,54^. Similarly, KAR CTD alterations could inhibit N-cadherin interaction and thereby influence synaptic compartmentalization and recruitment of KARs to the membrane^55^ and mutations disrupting C terminal PDZ ligand binding might influence secretory pathway processes, feedback systems and neuronal activity^20^. These synaptic-population and subcellular specific processes highlight the importance of KAR subunit availability and how variants, whether coding or acting through epigenetic mechanisms, could affect the KAR spatio-temporal patterns which may cause downstream alterations in the glutamate neurotransmission.

As with many NGS studies, a limitation of this study was that genomic coverage was dependent on read depth and quality of sequencing. Several regulatory UTR variants had to be excluded as they were not consistently called across cohorts and hence our results may have missed important regulatory KAR and NETOs alleles contributing to disease risk. For instance, we excluded an indel within the 3′UTR of *GRIK4* which we previously reported confers protection against developing bipolar disorder through altering GluK4 RNA and protein abundance^36,37^. Furthermore, intronic variants which were not assessed within *GRIK4* and *GRIK3* have also shown significantly association with response to antidepressant and antipsychotic medication^42,56–58^.

Case-control GWAS studies of individuals with schizophrenia have recently indicated common alleles within transcripts of the *C4* genes, which encodes a complement component 4 protein, and *SNAP25*, a vesicle fusion protein, contribute to risk of disease^59,60^. Both SNAP25 and members of a second complement cascade protein family (C1ql2 and C1ql3) are located at postsynaptic sites and bind to KAR subunits and thereby regulate KAR ion channel behaviour^61,62^. Further exploration of this emerging genetic risk pathway may aid in the development of new drugs to target neurodevelopmental conditions. Based on our findings, and with the need for translation from genetic risk factors to clear biomarkers of treatment response and disease prognosis, future studies using large population cohorts with collated phenotypic, genomic, medical and medication data (e.g. the UK biobank and USA ‘All of Us research program’) should involve the detailed characterisation of KAR and NETO risk alleles.

## Methods and Materials

Individuals with a clinical diagnosis of neurodevelopmental disorders were exome sequenced as part of the neurodevelopmental collections in the UK10K sequencing project (further details are provided in Supplementary Table S1). Ethical approval for genomic research and informed consent from all participants and/or their legal guardians was obtained previously by the UK10K consortium committee. The UK10K project was conducted in compliance with the Declaration of Helsinki statement of ethical principles. Access to the sequencing datasets was granted to Dr Knight under the UK10K Project access agreement ID5574. All individuals sequenced were of European ancestry.

In a first discovery phase, we examined approximately 846 individuals with schizophrenia or psychosis, 550 individuals with ASD, 124 individuals with intellectual disability, and individuals with a dual diagnosis of either ASD comorbid with ID (77) or psychosis comorbid with ID (175) (Fig. 1). The numbers assessed vary owing to exclusion of poor sequencing data within different KAR/NETO genes. In a second discovery phase, two additional schizophrenia cohorts, (the NEURO UKSCZ N = 553; and NEURO FSZNK N = 285) were investigated. Population controls came from the population control arms of the UK10K project (TwinsUK10K, Obesity UK10K; N = 2,095). In a follow-up phase of the study we attempted to replicate allelic associations by first comparing MAFs of variants identified in the case discovery cohorts with MAFs reported for the ExAC general population (N = 45,376). Subsequently, we compared alleles of interest in the psychiatric (N = 15,328) and non-psychiatric (N = 45,376) arms of the ExAC cohort.

Variant call files (VCF) were obtained from the European Genome-phenome Archive. VCF files for the non-psychiatric arm of ExAC was available from the ExAC website. The Genotype-Tissue Expression Project was used to identify primary transcripts expressed in brain and both brain-expressed and canonical transcripts were examined (Supplementary Table S2). A minimum of 12x read depth was accepted as a quality control. Functional annotation of coding variants was performed using snpEff, snpSift and dbNSFP. Variants were classified as: LoF variants (stop-gained, frameshift and splice-disrupting variants); missense; and regulatory (synonymous, non-damaging splicing site variants within 10 bp surrounding the exon and 3′UTR or 5′UTR variants). LoF annotation was conducted using the LoF Transcript Effect Estimator (LOFTEE, version 0.2). Mutation Taster, Panther DB, Align GVGD and PolyPhen2 were used to predict whether missense mutations were damaging. Splicing effects were assessed using the Human Splicing Finder (HSF 3.0). Minor allele frequencies (MAFs) were calculated for variants identified in the discovery phases and compared with MAFs derived from general population databases, e.g. GnoMAD. Variants were classified as common (MAF > 0.05), low frequency (MAF = 0.05–0.01), rare (MAF = 0.01–0.001) and ultra-rare (MAF = 0.001–0.0001).

Single allele association analysis was performed using either the Fisher’s exact test or the chi square tests and were two tailed. *P*-values were adjusted for correction using the Holm-Bonferroni method and significance was set at two levels; a genome wide level (*p* < 5 × 10^−8^) and a less conservative nominal level (*p* < 1 × 10^−6^). Odds Ratios (ORs) and confidence interval values were calculated using R software (v.3.4.1). Kernel methods SKAT and SKAT-O were implemented to identify genes carrying a significant burden of common, rare, and rare damaging variants^63^. Imputation of missing wild-type genotypes was conducted by using IMPUTE2 software^64^. Structural templates used for *in silico* protein modelling were acquired either from the Research Collaboratory for Structural Bioinformatics (RCSB) Protein Data Bank or generated from Uniprot amino acid sequences using RaptorX software^65^- further details are provided in Supplementary Table S3. *In silico* mutagenesis was performed using the mutagenesis function of Pymol (PyMOL Version 2.0, Schrödinger, LLC) and predicted hydrogen bonds within 8 A were examined in wild type and mutated structures. Free energy calculations (ΔG) were performed using FoldX v3.0^66^. The Adaptive Poisson-Boltzmann Solver (APBS) software package was used to study surface potential changes in the electrostatic surface potential in mutant proteins^67^.

Wild-type and mutant KARs (GluK2 K525E, GluK4 Y555N, and GluK4 L825W) were expressed in *Xenopus* oocytes using methods described previously^68^. *Xenopus* oocytes were supplied as ovarian lobes by the European *Xenopus* Resource Centre, University of Portsmouth, UK. Animal care and treatment were conducted in compliance with national and international laws and policies. The electrophysiology research protocol was performed in accordance with the University of Nottingham institutional guidelines and regulations. Human cDNA clones for GluK2 and GluK4 were obtained from GenScript (USA). Mutations were introduced into constructs using the QuikChange II Mutagenesis kit (Agilent Technologies) and cRNA generated using a mMessage mMachine kit (Invitrogen). Oocytes were injected with 50.6 nL cRNA (250–300 ng/μL) and incubated in GTP solution. Oocytes were perfused with frog Ringer solution at 10 mL/min and two-electrode voltage-clamped at −80 mV (Geneclamp 500, Axon Instruments). Glutamate was perfused at a flow rate of 10 mL/min at concentrations ranging between 10^−9^ M and 10^−3^ M for 10 s. Peak current amplitudes were normalised and plotted against glutamate concentration to determine EC_50_. Current decay time constants (τ) were estimated over the 10-s glutamate application by fitting exponential equations. Experiments were performed in multiple cells (n ≥ 5).

## Supplementary information

Supplementary Information

## Data Availability

The datasets generated during and/or analysed during the current study are not publicly available due access being granted to Dr Knight under the UK10K Project access agreement ID5574 but further information concerning identified variants is available from the corresponding author on reasonable request.
