# Supplementary material for: Damaging coding variants within kainate receptor channel genes are enriched in individuals with schizophrenia, autism and intellectual disabilities
Source: Sci Rep. 2019 Dec 16;9:19215. doi: 10.1038/s41598-019-55635-4 (PMC6915710; doi:10.1038/s41598-019-55635-4)
Supplement: Supplementary file 1 — Supplementary Information [file 41598_2019_55635_MOESM1_ESM.pdf]

Damaging coding variants within kainate receptor channel genes are enriched in individuals with schizophrenia, autism and intellectual disabilities.

Supplemental Information

Title: Damaging coding variants within kainate receptor channel genes are enriched in individuals with schizophrenia, autism and intellectual disabilities

Authors: Maria Koromina[1], Miles Flitton[1], Alix Blockley [1], Ian R. Mellor [1],

Helen M. Knight [1*]

[1] School of Life Sciences, University of Nottingham, Nottingham, NG7 2UH, UK

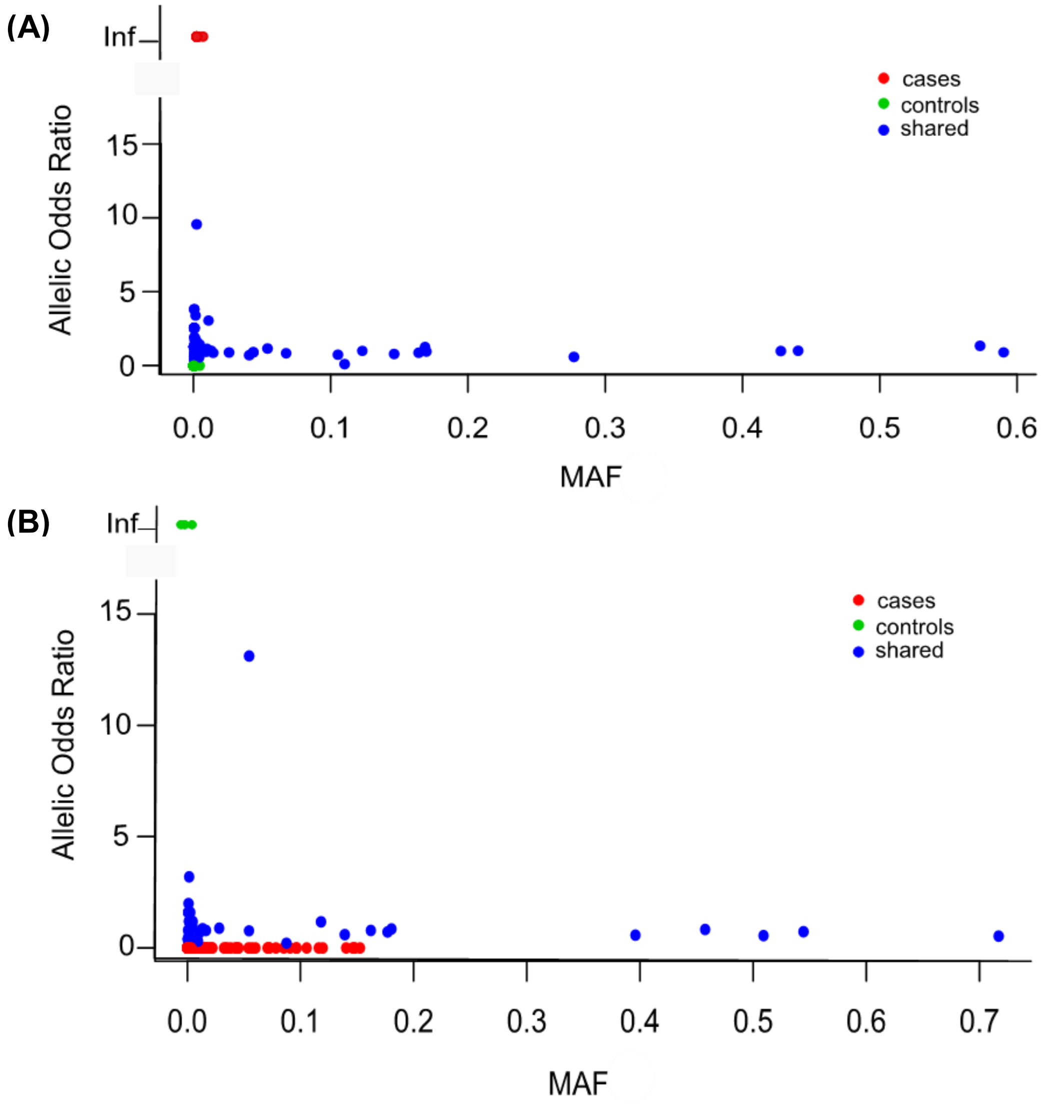

Figure S1. Allelic Odds Ratios plotted against minor allele frequencies of all variants identified in the first and second discovery phases. **(A)** Allelic odds ratios plotted against minor allele frequencies of coding variants identified in affected cases only, control individuals only or shared in cases and controls during the first phase study. **(B)** Allelic Odds Ratios plotted against minor allele frequencies of coding variants identified in affected cases only, control individuals only or shared in cases and controls during the second discovery study. Allelic ORs values are color coded with affected cases, red; controls, green; shared, blue. MAF: Minor Allele Frequency.

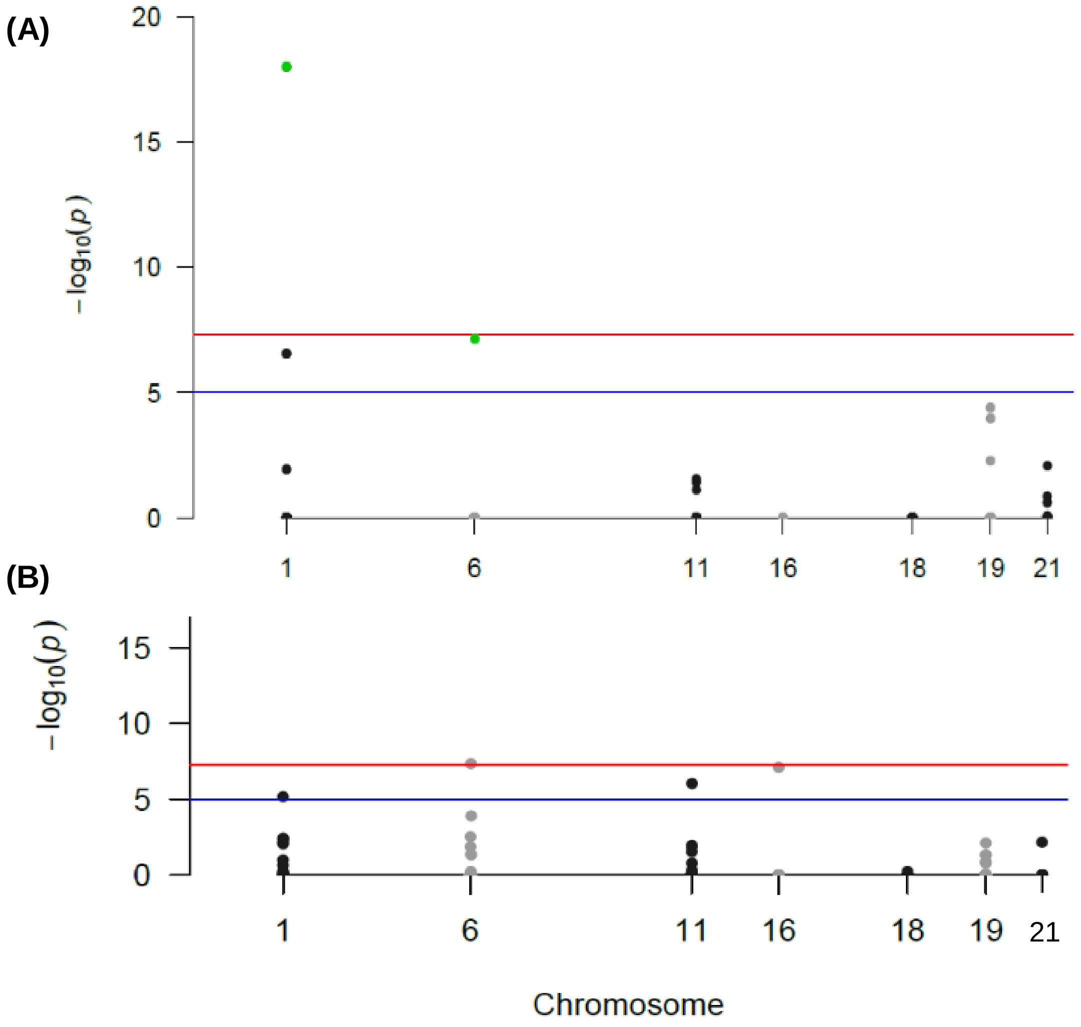

Figure S2. Manhattan plots showing significant associations for single alleles within *GRIK* and *NETO* genes. **(A)** *GRIK* and *NETO* variants identified in the first discovery phase. **(B)** *GRIK* and *NETO* variants identified in the second discovery schizophrenia cohort phase. Variants are plotted on the x-axis ordered by chromosomal position and *P* -values are plotted as -log(*p*) on the y axis. Variants that achieved genome-wide significance ($p < 5 \times 10^{-8}$) are highlighted in green. The blue line depicts a suggestive significance threshold.

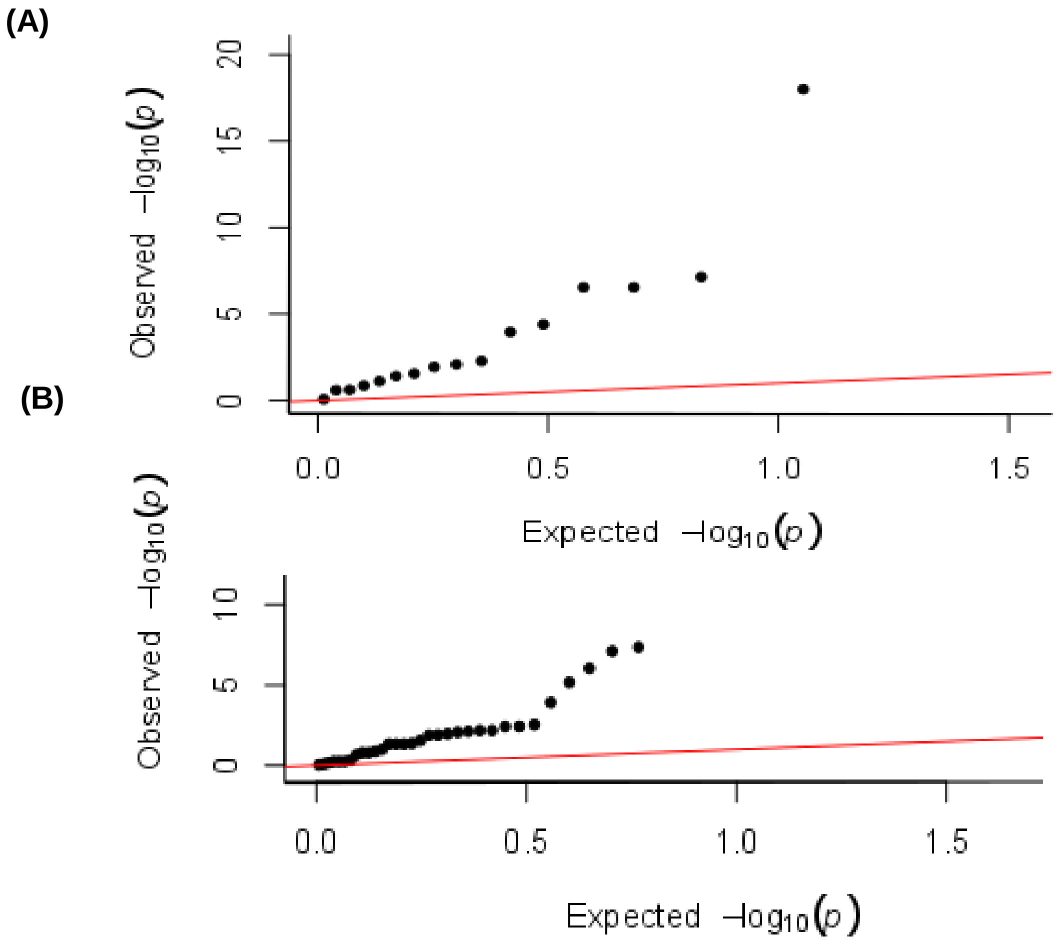

Figure S3. QQ plots showing the observed significance of associations plotted as -log (*p*) against the expected p values. **(A)** GRIK and NETO coding variants from the initial discovery phase. **(B)** GRIK and NETO coding variants from the schizophrenia replication cohort. A Bonferroni correction was applied to all *p* values. In both QQ plots an early separation of the observed from the expected *p* values is shown suggesting true disease risk associations.

(A)

(B)

(C)

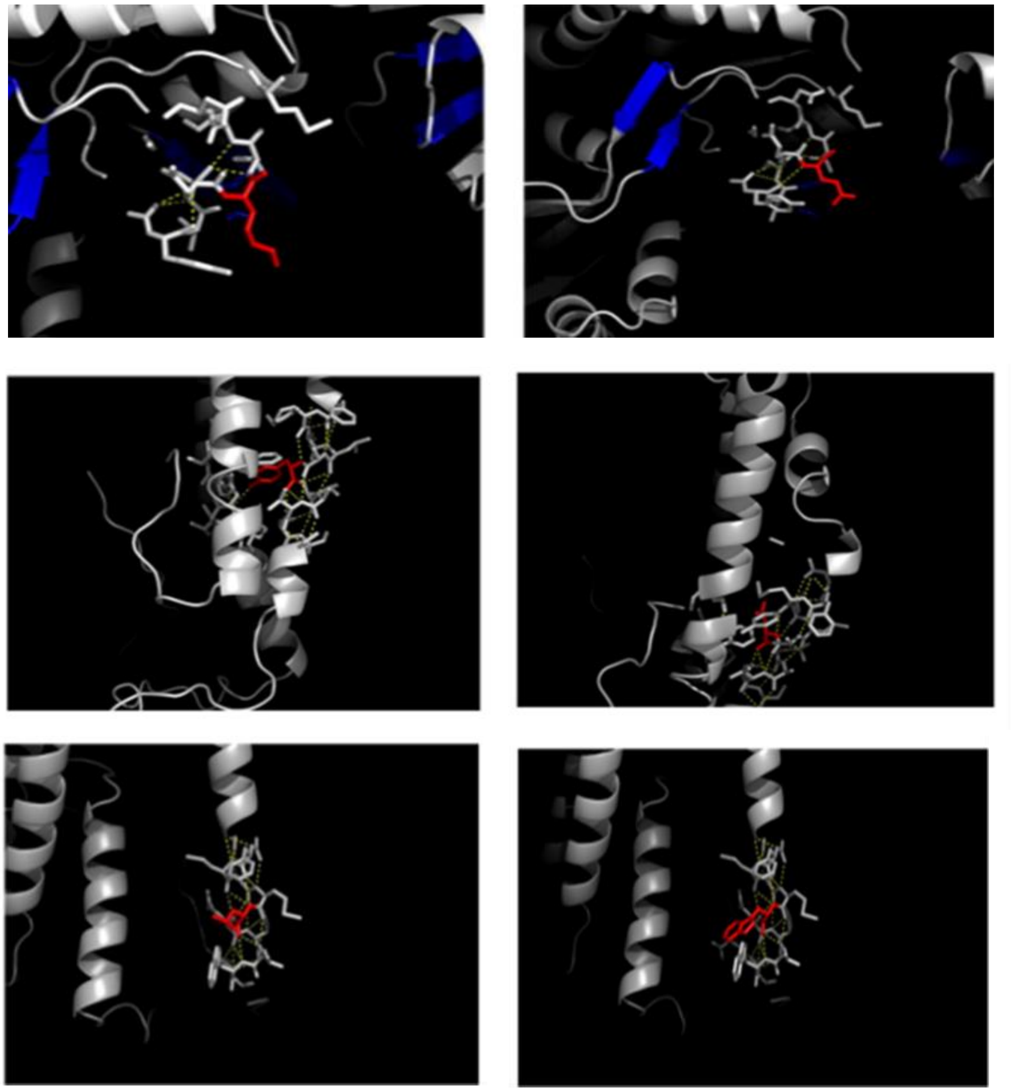

Figure S4. Protein modelling of three missense variants identified within GluK2 and GluK4 subunits. **(A)** GluK2 K525E is located in the ligand binding domain and leads to creation of a hydrogen bond. **(B)** GluK4 Y555N is located in the first transmembrane domain (TMD1) and causes the disruption of a hydrogen bond. **(C)** GluK4 L825W is located in the last transmembrane domain (TMD3) and does not cause a change in hydrogen bonds.

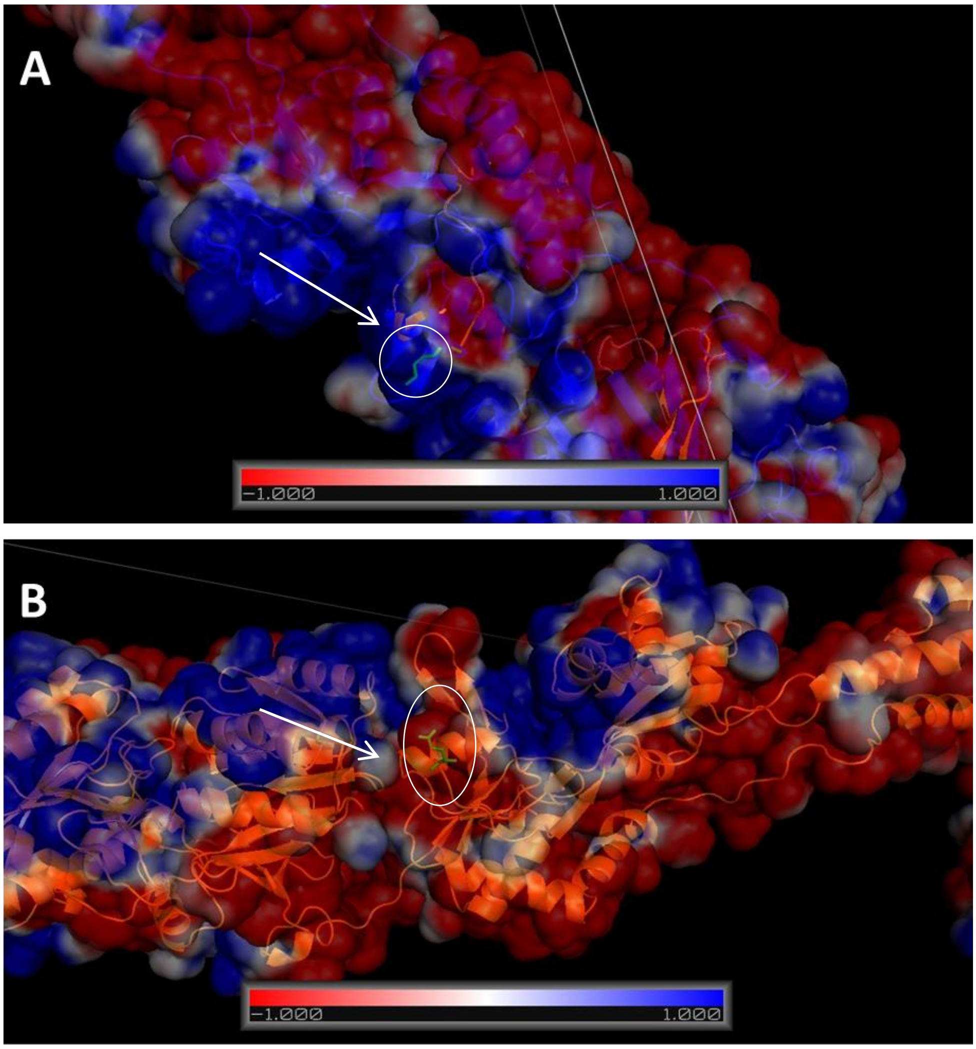

Figure S5. Protein modelling of the surface electrostatic potential for GluK2 K525E damaging missense variant. The green stick denotes GluK2 K525E (white arrow). Blue regions depict a positive electrostatic potential and red regions a negative electrostatic potential. **(A)** Shows the electrostatic potential of wildtype GluK2 K525 and **(B)** the electrostatic potential of mutated GluK2 E525. A decrease in positive electrostatic potential was observed for the mutated allele.

**Datasets**	**Seq. context**	**Depth**	**No.**	**Disease**	**Sample acknowledgements**
UK10K_RARE_FIND (124) main release	Exome	>40x	124	ID	The Familial Intellectual Disability study or FIND study
UK10K_NEURO_ASD_GALLAGHER	Exome	<10x	77	ASD with ID	Trinity College Dublin Autism Genetics Collection
UK10K_NEURO_ASD_SKUSE	Exome	>40x	341	ASD	Institute of Child Health & Great Ormond Street Hospital Autism Families Study
UK10K_NEURO_IOP_COLLIER	Exome	~20x	172	SCZ, BP, Psy	See list of publications (*)
UK10K_NEURO_MUIR	Exome	>50x	175	SCZ, ASD, Psy with ID	Edinburgh MR-psychosis samples
UK10K_NEURO_EDINBURGH	Exome	>50x	234	SCZ	Edinburgh Schizophrenia samples
UK10K_NEURO_ABERDEEN	Exome	>50x	392	SCZ	Scottish schizophrenia cases
UK10K_NEURO_GURLING	Exome	>40x	48	SCZ	University College London Schizophrenia Family Samples
UK10K_COHORT_IMGSAC	Exome	45x	113	ASD	"The International Molecular Genetic Study of Autism Consortium (IMGSAC)"
UK10K_COHORT_MGAS	Exome	45x	97	ASD	The Molecular Genetics of Autism Study
UK10K_NEURO_UKSCZ	Exome	50x	553	SCZ	Cardiff Scz
UK10K_NEURO_FSZNK	Exome	30x	285	SCZ	National Institute for Health and Welfare (THL) Finnish Schizophrenia. Families from the "The genetic etiology of severe mental disorders in Finland" study
UK10K_OBESITY_TWINSUK	Exome	>30x	430*	Control Population	Generation Scotland:Scottish Family Health Study (GS:SFHS)
UK10K_COHORT_TWINSUK	Whole genome	<12x	1854	Control Population	The TwinsUK Cohort

Table S1. UK10K datasets used in the current study. Dataset names, sequencing context and depth coverage, number of individuals, disease diagnosis and sample acknowledgements are presented. ASD, Autism Spectrum Disorder; BP, Bipolar Disorder; BMI, Body Mass Index; ID, Intellectual Disability; Psy, psychosis; Scz, schizophrenia; Seq. Context, sequencing context.

***Maudsley family study** Distribution of symptom dimensions across Kraepelinian divisions. Dikeos DG, Wickham H, McDonald C, Walshe M, Sigmundsson T, Bramon E, Grech A, Toulopoulou T, Murray R, Sham PC. Br J Psychiatry. 2006 Oct;189:346-53.
***GAP study** High-potency cannabis and the risk of psychosis. Di Forti M, Morgan C, Dazzan P, Pariante C, Mondelli V, Marques TR, Handley R, Luzi S, Russo M, Paparelli A, Butt A, Stilo SA, Wiffen B, Powell J, Murray RM. Br J Psychiatry. 2009 Dec;195(6):488-91.
***The Maudsley Twin Study.** Genetic overlap between episodic memory deficits and schizophrenia: results from The Maudsley Twin Study. Owens SF, Picchioni MM, Rijsdijk FV, Stahl D, Vassos E, Rodger AK, Collier DA, Murray RM, Toulopoulou T. Psychol Med. 2011 Mar;41(3):521-32

Gene	HGNC	Transcript code	Protein
GRIK1	4579	ENST00000327783	E7ENK3
GRIK2	4580	NM_021956	NP_068775
GRIK3	4581	NM_000831	NP_000822
GRIK4	4582	NM_01282470	NP_001269399
GRIK5	4583	NM_002088	NP_002079
NETO1	13823	NM_001201465	NP_001188394
NETO2	14644	NM_001201477	NP_00188406

Table S2. Gene, Hugo Gene Nomenclature Committee (HGNC), gene code, transcript and protein codes of *GRIK* and *NETO* genes. The transcripts were identified as the primary transcripts expressed in brain using the Genotype-Tissue Expression (GTEx) database.

Damaging coding variants within kainate receptor channel genes are enriched in individuals with schizophrenia, autism and intellectual disabilities.

Protein	PDB name	Source
GluK1	-	RaptorX
GluK1 LBD dimer	2ZNS	PDB
GluK2	-	RaptorX
GluK2 LBD dimer	2XXT	PDB
GluK2EM LBD dimer	5CMM	PDB
GluK3	-	RaptorX
GluK4	-	RaptorX
GluK4 LBD	5IKB	PDB
GluK5	-	RaptorX
NETO1	-	RaptorX
NETO2	-	RaptorX

Table S3. Protein modelling templates and Protein Data Bank (PDB) files used in the *in silico* protein modeling. The PDB name and source of template is listed for each protein.

Gene	Total variants	LoF & missense variants	Rare LoF & missense variants	Regulatory variants	Rare regulatory variants
GRIK1	42	24	24	18	16
GRIK2	38	21	18	17	14
GRIK3	71	25	23	46	45
GRIK4	55	27	25	28	24
GRIK5	47	31	26	16	14
NETO1	26	14	13	12	10
NETO2	18	12	8	6	5
Total	297	154	137	143	128

Table S4. Coding variants within GRIK and NETO genes identified during the first discovery phase. The absolute numbers of total, LoF and missense, rare LoF and missense, and, regulatory variants are detailed.

Gene	Total variants	LoF & missense variants	Rare LoF & missense variants	Regulatory variants	Rare regulatory variants
GRIK1	8	4	4	4	4
GRIK2	8	5	5	3	3
GRIK3	9	4	4	5	5
GRIK4	12	7	7	5	5
GRIK5	9	5	5	4	4
NETO1	12	5	5	7	7
NETO2	8	6	6	2	2
Total	66	36	36	30	30

Table S5. Summary of coding variants within GRIK and NETO genes identified exclusively within affected individuals during the first discovery phase. The absolute numbers of total, LoF and missense, rare LoF and missense, and, regulatory variants are detailed.

Damaging coding variants within kainate receptor channel genes are enriched in individuals with schizophrenia, autism and intellectual disabilities.

Gene	Total variants	LoF & missense variants	Rare LoF & missense variants	Regulatory variants	Rare regulatory variants
GRIK1	24	16	16	8	8
GRIK2	20	11	10	9	9
GRIK3	40	17	15	23	23
GRIK4	28	15	13	13	13
GRIK5	31	21	20	10	10
NETO1	7	4	4	3	3
NETO2	8	5	1	3	3
Total	158	89	79	69	69

Table S6. Summary of coding variants within GRIK and NETO genes identified exclusively within control individuals during the first discovery phase. The absolute numbers of total, LoF and missense, rare LoF and missense, and, regulatory variants are detailed.

Gene	Total variants	LoF & missense variants	Rare LoF & missense variants	Regulatory variants	Rare regulatory variants
GRIK1	10	4	4	6	1
GRIK2	10	5	3	5	2
GRIK3	22	4	4	18	12
GRIK4	15	5	5	10	10
GRIK5	8	6	1	2	2
NETO1	7	5	4	2	2
NETO2	2	1	1	1	0
Total	74	30	22	44	29

Table S7. Summary of coding variants within GRIK and NETO genes shared within control and case individuals during the first discovery phase. The absolute numbers of total, LoF and missense, rare LoF and missense, and, regulatory variants are detailed.

	PD/PsD	Benign
**Cases**	**77**	**13**
**Controls & Shared**	**38**	**22**

Table S8. Number of damaging missense or benign missense variants identified in affected cases only or shared or in controls only during the first discovery phase. Abbreviations: PD, probably damaging; PsD, possibly damaging.

Damaging coding variants within kainate receptor channel genes are enriched in individuals with schizophrenia, autism and intellectual disabilities.

	**All MAF All 'of interest'**	**All MAF LoF & Mis**	**All MAF Reg**	**MAF < 1% All 'of interest'**	**MAF < 1% LoF & Mis**	**MAF < 1% Reg**
GRIK1	p=6.13x10^{-4}	p=0.003	p=0.023	p=0.340	p=0.548	p=0.273
GRIK2	p= 0.022	p=0.427	p=0.024	p=0.006	p= 0.021	p= 0.144
GRIK3	p= 1.26x10^{-5}	p=0.018	p= 0.002	p=0.003	p= 0.549	p= 0.002
GRIK4	p= 1.9x10^{-4}	p= 0.008	p= 0.012	p= 1.99x10^{-4}	p= 0.008	p= 0.012
GRIK5	p=3.96x10^{-6}	**p= 9.99x10^{-6} ***	p= 0.020	p= 4.07x10^{-6}	**p= 9.99x10^{-6} ***	p= 0.021
NETO1	**p= 8.95x10^{-7} ***	p= 0.154	**p= 4.66x10^{-16} ***	p= 0.06	p= 0.154	p= 0.055
NETO2	p= 0.852	p= 0.385	p= 0.80	p= 0.514	p= 0.385	p= 0.799
ALL GENES	**p= 3.38x10^{-20} ***	**p= 2.97x10^{-8} ***	**p=7.72 x10^{-8} ***	**p= 2.07x10^{-15} ***	**p= 6.02x10^{-7} ***	p= 1.17x10^{-6}

Table S9. Burden analysis results for the first discovery phase and at an individual gene level. Tests were conducted first across all variant categories and including all MAFS, followed by LoF and missense, regulatory and rare variants (MAF < 1%) analysed separately. The asterisk (*) denotes variant categories showing GWA or nominal significance association p-values.

Damaging coding variants within kainate receptor channel genes are enriched in individuals with schizophrenia, autism and intellectual disabilities.

	All MAF All 'of interest'	All MAF Mis & LoF	All MAF Reg	MAF < 1% All 'of interest'	MAF < 1% LoF & Mis	MAF <1% Reg
GRIK1	*p*= 0.17	*p*= 0.33	*p*= 0.15	*p*= 0.67	*p*= 0.62	*p*= 0.58
GRIK2	*p*= 0.03	*p*= 0.18	*p*= 0.03	*p*= 0.30	*p*= 0.04	*p*= 0.87
GRIK3	*p*= 0.05	*p*= 0.30	*p*= 0.02	*p*= 0.08	*p*= 0.18	*p*= 0.02
GRIK4	*p*= 0.05	*p*= 0.47	*p*= 0.08	*p*= 0.01	*p*= 0.33	*p*= 0.05
GRIK5	**$p = 3.12 \times 10^{-8}$ ***	**$p = 7.83 \times 10^{-10}$ ***	*p*= 0.01	**$p = 2.10 \times 10^{-10}$ ***	**$p = 7.83 \times 10^{-10}$ ***	$p = 2.00 \times 10^{-4}$
NETO1	**$p = 6.76 \times 10^{-6}$ ***	$p = 4 \times 10^{-4}$	$p = 2.64 \times 10^{-5}$	**$p = 5.44 \times 10^{-6}$ ***	$p = 1 \times 10^{-4}$	$p = 1.84 \times 10^{-5}$
NETO2	*p*= 0.53	*p*= 0.49	*p*= 0.28	*p*= 0.83	*p*= 0.49	*p*= 0.44

Table S10. Burden analysis data for each individual gene and for a psychosis phenotype generated during for the first discovery phase. Individuals diagnosed with psychosis were compared to unaffected control individuals. Tests were conducted first across all variant categories and including all MAFS, followed by LoF and missense, regulatory and rare variants (MAF < 1%) analysed separately. The asterisk (*) denotes variant categories showing GWA or nominal significance association p-values.

Damaging coding variants within kainate receptor channel genes are enriched in individuals with schizophrenia, autism and intellectual disabilities.

	All MAF All 'of interest'	All MAF LoF & Mis	All MAF Reg	MAF < 1% All 'of interest'	MAF < 1% LoF & Mis	MAF < 1% Reg
GRIK1	p= $1.20x10^{-5}$	p=$2x10^{-4}$	p= 0.005	p= 0.112	p= 0.418	p= 0.105
GRIK2	p= 0.003	p= 0.165	p= 0.074	p= $3x10^{-4}$	p= 0.064	p= $3.00x10^{-4}$
GRIK3	**p= $3.31x10^{-13}$ ***	**p= $2.33x10^{-7}$ ***	p= 0.003	p= 0.016	p= 0.535	p= 0.003
GRIK4	p= $4x10^{-4}$	p= 0.005	p= $4.09x10^{-5}$	**p= $7.89x10^{-9}$ ***	**p= $3.07x10^{-6}$ ***	p= 0.017
GRIK5	p= 0.076	p= 0.130	p= 0.492	p= 0.002	p= 0.130	p= 0.005
NETO1	**p= $2.79x10^{-12}$ ***	p= 0.390	p= 0.328	p= 0.086	p= 0.074	p= 0.328
NETO2	p= 0.697	p= 0.299	p=0.692	p= 0.420	p=0.299	p= 0.439

Table S11. Burden analysis data generated for ASD and ID phenotypes within individual genes during the first discovery phase. Tests were conducted across all variant categories and included variants at all MAFs followed by LoF and missense, regulatory and rare variants (MAF < 1%) analysed separately. The asterisk (*) denotes variant categories showing GWA or nominal significance association p-values.

Damaging coding variants within kainate receptor channel genes are enriched in individuals with schizophrenia, autism and intellectual disabilities.

Gene	Total variants	LoF & missense variants	Rare LoF & missense variants	Regulatory variants	Rare regulatory variants
GRIK1	23	12	11	11	8
GRIK2	28		10	14	10
GRIK3	44	23	22	21	20
GRIK4	28	10	7	18	14
GRIK5	31	17	17	14	12
NETO1	28	13	7	15	11
NETO2	15	8	6	7	7
Total	197	97	80	100	82

Table S12. Summary of coding variants within GRIK and NETOs genes identified during the second discovery phase. The absolute numbers of total, LoF and missense, rare LoF and missense, and, regulatory variants are detailed.

	Total variants	LoF & missense variants	Rare LoF & missense variants	Regulatory variants	Rare regulatory variants
GRIK1	8	4	4	4	4
GRIK2	8	5	5	3	3
GRIK3	9	4	4	5	5
GRIK4	12	7	7	5	5
GRIK5	9	5	5	4	4
NETO1	12	5	5	7	7
NETO2	8	6	6	2	2
Total	66	36	36	30	30

Table S13. Summary of coding variants within GRIK and NETOs genes identified exclusively within affected individuals during the second discovery phase. The absolute numbers of total, LoF and missense, rare LoF and missense, and, regulatory variants are detailed.

Damaging coding variants within kainate receptor channel genes are enriched in individuals with schizophrenia, autism and intellectual disabilities.

	Total variants	LoF & missense variants	Rare LoF & missense variants	Regulatory variants	Rare regulatory variants
GRIK1	11	4	5	7	4
GRIK2	13	6	2	7	6
GRIK3	30	17	17	13	12
GRIK4	6	2	0	4	5
GRIK5	14	9	9	5	7
NETO1	8	4	0	4	3
NETO2	6	2	0	4	4
Total	88	44	32	44	41

Table S14. Summary of coding variants within GRIK and NETOs genes found exclusively within control individuals found identified during the second discovery phase. The absolute numbers of total, LoF and missense, rare LoF and missense, and, regulatory variants are detailed.

	Total variants	LoF & missense variants	Rare LoF & missense variants	Regulatory variants	Rare regulatory variants
GRIK1	4	4	2	0	0
GRIK2	7	3	2	4	1
GRIK3	5	2	1	3	2
GRIK4	10	1	0	9	3
GRIK5	8	3	3	5	2
NETO1	8	4	2	4	3
NETO2	1	0	0	1	1
Total	43	17	10	26	11

Table S15. Summary of coding variants within GRIK and NETOs genes found shared within case and control individuals identified during the second discovery phase. The absolute numbers of total, LoF and missense, rare LoF and missense, and, regulatory variants are detailed.

Damaging coding variants within kainate receptor channel genes are enriched in individuals with schizophrenia, autism and intellectual disabilities.

	PD /PsD	Benign
**Cases**	**34**	**14**
**Controls & Shared**	**24**	**26**

Table S16. Number of damaging missense or benign missense variants identified in affected cases only or shared or in controls only during the second discovery phase. Abbreviations: PD, probably damaging; PsD, possibly damaging.

	**All MAF, All 'of interest'**	**All MAF, Reg**	**All MAF, LoF & mis**	**MAF < 1%, All 'of interest'**	**MAF < 1%, Reg**	**MAF < 1%, LoF & mis**
GRIK1	p= 0.55	p= 0.17	p= 0.51	p= 0.26	p= 0.139	p= 0.37
GRIK2	p= 0.22	p= 0.01	p= 0.26	p= 0.01	p= 0.02	p= 0.46
GRIK3	**p= 2.17x10^{-11} ***	p= 0.02	**p= 5.24x10^{-10} ***	p= 0.01	p= 6.48x10^{-5}	p= 0.02
GRIK4	p= 0.01	p= 1x10^{-4}	p= 0.26	**p= 3.57x10^{-12} ***	p= 2x10^{-4}	p= 0.35
GRIK5	p= 0.02	p= 0.02	p= 0.13	p= 0.01	p= 3.37x10^{-5}	p= 0.13
NETO1	**p= 1.73x10^{-10} ***	**p= 1.61x10^{-28} ***	p= 0.28	p= 0.02	p= 0.01	p= 0.17
NETO2	**p=1.48x10^{-9} ***	**p= 8.03x10^{-10} ***	p= 0.37	p= 0.31	p= 0.02	p= 0.37

Table S17. Burden analysis data at an individual gene level generated during the second discovery phase. Individuals diagnosed with psychosis were compared to unaffected control individuals. Tests were conducted across all variant categories and included variants at all MAFs. The asterisk (*) denotes variant categories showing GWA or nominal significance association p-values.

Damaging coding variants within kainate receptor channel genes are enriched in individuals with schizophrenia, autism and intellectual disabilities.

Gene	cDNA	Type	Dataset	Allele count cases	MAF cases	Allele count non-psy arm	MAF non-psy arm	*P* value	Odds ratio
GRIK1	c.1173C>T (p.Asp391Asp)	Syn	All neuro (1,700 exomes)	434/ 3,288	0.132	17,203/ 90,756	0.23	2.41×10^{-16} *	0.65
GRIK2	c.-88A>G	Splice	Replication SCZ (838 exomes)	4/ 1,662	0.0024	0/ 90,756	0	2.42×10^{-6}	Inf
GRIK2	c.1095+7T>C	Splice	Replication SCZ (838 exomes)	243/ 1,218	0.1995	24,055/ 90,756	0.36	1.93×10^{-17} *	0.55
GRIK3	c.357T>C (p.Asn119Asn)	Syn	All neuro (1,700 exomes)	15/ 3,288	0.0046	3,550/ 90,756	0.04	2.72×10^{-35} *	0.11
GRIK4	c.1582G>A (p.Val528Ile)	Mis	All neuro (1,700 exomes)	8/ 3,288	0.0024	1,028/ 90,756	0.01	1.09×10^{-6}	0.21
GRIK4	c.1635G>A (p.Pro545Pro)	Syn	Replication SCZ (838 exomes)	556/ 1,218	0.4565	27,872/ 90,756	0.44	4.35×10^{-22} *	1.80
GRIK5	c.2679C>G (p.Ala893Ala)	Syn	All neuro (1,700 exomes)	11/ 3,288	0.0033	0/ 90,756	0	4.56×10^{-15} *	Inf
GRIK5	c.1843C>T (p.Leu615Leu)	Syn	Replication SCZ exomes (838 exomes)	6/ 1,218	0.0018	15/ 90,756	0.0002	6.16×10^{-5}	21.9
NETO1	c.1460C>G (p.Ala487Gly)	Mis	All neuro (1,700 exomes)	3/ 3,288	0.0009	704/ 90,756	0.008	1.12×10^{-6}	0.11
NETO1	c.-41G>A	Splice	Replication SCZ exomes (838 exomes)	4/ 3,288	0.0012	0/ 90,756	0	2.40×10^{-6}	Inf
NETO2	c.1366T>A (p.Ser456Thr)	Mis	All neuro (1,700 exomes)	3/ 3,288	0.0009	808/ 90,756	0.009	3.47×10^{-8}	0.10

Table S18. Re-assessing single allele associations using the EXAC non-psy control population. Damaging missense variants with nominal significant associations and synonymous or splice site variants with GWA or nominal significant associations are displayed. Gene, Amino acid change, variant cDNA and HGVS nomenclature, case and control minor allele frequencies, *p*

values and odds ratio are provided. The asterisk before the p values (*) indicates genetic variants with significant GWA associated p values. Abbreviations: EXAC, Exome Aggregation Consortium; Inf, infinity; MAF, Minor Allele Frequency; Mis, missense; protein cons, protein consequence; Scz, schizophrenia; Splice, splice site variant; Syn, synonymous.

Damaging coding variants within kainate receptor channel genes are enriched in individuals with schizophrenia, autism and intellectual disabilities.

Gene	cDNA	Type	MAF (count) psy ExAC	MAF (count) control ExAC	*P* value	OR (CI)
GRIK1	c.1173C>T (p.Asp391Asp)	Syn	0.145 (4,354/30,000)	0.189 (17,203/90,756)	6.135×10^{-68}	0.726 (0.700 – 0.753)
GRIK2	c.1095+7T>C	Spl	0.219 (6,580/30,000)	0.265 (24,055/90,756)	4.496×10^{-56}	0.779 (0.755 – 0.804)
GRIK3	c.357T>C (p.Asn119Asn)	Syn	0.013 (392/30,000)	0.039 (3 558/90,756)	7.02×10^{-108}	0.325 (0.291 – 0.361)
GRIK3	c.2593A>G (p.Arg865Gly)	Mis	0.006 (190/30,000)	0.004 (396/90,756)	2.075×10^{-5}	1.491 (1.216 – 1.734)
GRIK4	c.1635G>A (p.Pro545Pro)	Syn	0.348 (10,433/30,000)	0.307 (27 872/90,756)	2.597×10^{-39}	1.212 (1.170 – 1.236)
GRIK5	c.1843C>T (p.Leu615Leu)	Syn	0.000 (0/30,000)	0.0002 (15/90,756)	0.026	0.000 (0.000 – 0.843)

Table S19. Re-assessing single allele associations using ExAC case and control data. Damaging missense variants with nominal significant associations and synonymous or splice site variants with GWA or nominal significant associations are displayed. Abbreviations: CI, Confidence Interval; ExAC, Exome Aggregation Consortium; MAF, Minor Allele Frequency; Mis, missense variant; OR, Odds Ratio; psy, psychiatric cases; Spl, splice variant; Syn, synonymous variant.

Receptor	[Agonist] (M)	I_{peak} (nA) - Glu	(τ_{decay}) (ms) - Glu	τ_{deact} (s) - Glu
hGluK2	10^{-4}	-154 ± 95.5 (12)	805 ± 220 (11)	1.5 ± 0.8 (7)
	10^{-3}	-306 ±189.1 (12)	517 ± 141 (14)	2.3 ± 0.8 (7)
hGluK2/hGluK4	10^{-4}	-60.0 ± 69.4 (12)	354 ± 42 (11)	
	10^{-3}	-56.6 ± 50.8 (12)	296 ± 48 (10)	
hGluK2/hGluK4 Y555N	10^{-4}	-13.3 ± 14.8 (13)	810 ±127 (10)	
	10^{-3}	-34.4 ± 59 (13)	343 ± 56 (11)	
hGluK2/hGluK4 L825W	10^{-4}	-22.0 ± 24.4 (17)	801 ± 240 (8)	
	10^{-3}	-38.0 ± 39.4 (18)	318 ± 60 (9)	
hGluK2/hGluK2 K525E	10^{-4}	-16.2 ± 15 (16)	478 ± 80 (5)	3.4 ± 1.0 (7)
	10^{-3}	-89.0 ± 64 (17)	206 ± 51 (6)	6.0 ± 2.9 (8)

Table S20. Time constants for current decay (τ_{decay}) and deactivation (τ_{deact}) and peak current measurements (I_{peak}) are presented for wild-type and mutated h.GluK2 and h.GluK2/GluK4 KARs with SEM and N values for two standard agonist (glutamate) concentrations. hGluK2 and hGluK2 denotes human GluK2 and GluK4 subunits.
